# New eye tracking metrics system: the value in early diagnosis of autism spectrum disorder

**DOI:** 10.3389/fpsyt.2024.1518180

**Published:** 2024-12-11

**Authors:** Raymond Kong Wang, Kenneth Kwong, Kevin Liu, Xue-Jun Kong

**Affiliations:** ^1^ Department of Radiology, Massachusetts General Hospital, Harvard Medical School, Boston, MA, United States; ^2^ Department of Mathematics and Statistics, University of Massachusetts Lowell, Lowell, MA, United States; ^3^ Department of Psychiatry, Beth Israel Deaconess Medical Center, Harvard Medical School, Boston, MA, United States

**Keywords:** autism spectrum disorder (ASD), eye tracking (ET), area of interest (AOI), gaze abnormality, early diagnosis

## Abstract

**Background:**

Eye tracking (ET) is emerging as a promising early and objective screening method for autism spectrum disorders (ASD), but it requires more reliable metrics with enhanced sensitivity and specificity for clinical use.

**Methods:**

This study introduces a suite of novel ET metrics: Area of Interest (AOI) Switch Counts (ASC), Favorable AOI Shifts (FAS) along self-determined pathways, and AOI Vacancy Counts (AVC), applied to toddlers and preschoolers diagnosed with ASD. The correlation between these new ET metrics and Autism Diagnostic Observation Schedule, Second Edition (ADOS-2) scores via linear regression and sensitivity and specificity of the cut-off scores were assessed to predict diagnosis.

**Results:**

Our findings indicate significantly lower FAS and ASC and higher AVC (P<0.05) in children with ASD compared to their non-ASD counterparts within this high-risk cohort; the significance was not seen in total fixation time neither pupil size (p > 0.05). Furthermore, FAS was negatively correlated with ADOS-2 total scores and social affect (SA) subscale (p < 0.05). Among these new ET metrics, AVC yielded the best sensitivity 88-100% and specificity 80-88% with cut off score 0.305-0.306, followed by FAS and ASC to separate ASD from non-ASD for diagnosis.

**Conclusions:**

This study confirms the utility of innovative ET metrics—FAS, AVC, and ASC—which exhibit markedly improved sensitivity and specificity, enhancing ASD screening and diagnostic processes.

## 1 Introduction

Early diagnosis and intervention are pivotal in determining long-term outcomes for individuals with Autism Spectrum Disorder (ASD). The prognostic implications underscore the necessity for developing readily accessible and effective early detection tools (1–4). Current diagnostic frameworks such as the Diagnostic and Statistical Manual of Mental Disorders, Fifth Edition (DSM-5) (5), and the Autism Diagnostic Observation Schedule, Second Edition (ADOS-2) (6),provide structured criteria for early diagnosis. Nevertheless, these conventional methods are often elaborate, time-consuming, and resource-intensive. This complexity can delay diagnostic and therapeutic interventions, particularly in underserved populations (7, 8). Despite recent advances in diagnostic methodologies, the mean age of diagnosis persists at four to five years (4). Furthermore, disparities in diagnosis times are evident, with ethno-racial minorities and non-English speaking children diagnosed significantly later than their white counterparts (9). Addressing these disparities is critical, underscoring the urgent need for innovative diagnostic tools that facilitate earlier detection and intervention in high-risk populations.

Amidst the subjective limitations of standard assessments, recent research has shifted towards objective biomarkers for ASD diagnosis. Eye tracking (ET) technology has gained prominence as a promising diagnostic tool due to its inherent objectivity and rapid assessment capabilities (10–14). Traditionally utilized in human perception studies and extensively in ASD research, ET technology quantifies eye positions, movements (11, 15), and pupil size dynamics (16–18) to delineate zones of user interest. Distinctive eye movement patterns and gaze behaviors in ASD, such as challenges in interpreting gaze cues, a preference for systematically arranged images over faces, and a lack of right hemispheric dominance for facial processing, have been well-documented (19–22). Essential metrics in ASD ET research include Total Gaze Count (TGC) and Total Fixation Time (TFT), which respectively measure the frequency of gazes and the duration of eye fixation within designated Areas of Interest (AOIs) (11–14). Recent investigations have highlighted significant reductions in TFT across most AOIs for ASD subjects compared to controls in developmental cohorts, reinforcing the diagnostic potential of these parameters (13).

Despite the promising aspects of ET, variability in its protocols and paradigms has historically limited its utility as a consistent diagnostic tool (23, 24). It is important to note that traditionally used ET metrics such as TFT have limited sensitivity and specialty and are unable to identify subtle changes and recent studies have reported newly introduced metrics that can achieve higher accuracies for diagnosis (25). Thus, the present study aims to introduce a set of novel ET metrics such as Area of Interest Switch Counts (ASC), Favored Area of Interest Shifts (FAS), and Area of Interest Vacancy Counts (AVC) with the hypothesis that AVC and the adjunct metrics are able to provide improved accuracy and specificity in the discriminability between ASD and non-ASD cohorts. These metrics are designed to quantify dynamic shifts between AOIs and are hypothesized to reflect fundamental ASD-related deficits such as joint attention, social referencing, and theory of mind. Integrating these new metrics with established diagnostic assessments like ADOS-2, we aim to significantly enhance the specificity and sensitivity of ET for ASD diagnosis. Preliminary findings have facilitated the identification of optimal cutoff scores, providing foundational proof-of-concept and methodologies poised to refine ASD diagnostic approaches through advanced ET metrics.

## 2 Methods

### 2.1 Participants

This study involved thirty-nine individuals aged 18 to 84 months, identified as high-risk for ASD by clinicians or caregivers in Massachusetts and its surrounding states. High-risk status was confirmed via telephone screening prior to enrollment. Inclusion criteria required participants to meet one or more of the following: (1) having at least one sibling with a clinical ASD diagnosis; (2) caregiver or clinician concerns regarding the child’s development in social interaction, play, or other behaviors; (3) scoring in the positive range on the Modified Checklist for Autism in Toddlers (M-CHAT). Participants with major congenital or genetic disorders, or behavioral issues likely to cause significant stress during testing were excluded. Those previously diagnosed with ASD were included without disclosing their diagnosis to the examiner. Subjects were categorized into ASD and non-ASD groups based on DSM-5 criteria, evaluated by two field experts.

### 2.2 Assessment instruments and protocols

#### 2.2.1 Ethics and informed consent

The study was conducted according to the guidelines of the Declaration of Helsinki and approved by the Institutional Review Board of Massachusetts General Hospital 2017P001667, 13 July 2018). The secondary use of research samples/data was approved by the Institutional Review Board of Massachusetts General Hospital (2020P004102; January 7, 2021). Written informed consent was obtained from parents or guardians of all subjects involved in the study.

#### 2.2.2 Eye tracking setup

Eye tracking data were collected using a Tobii X3-120 eye tracker, with the screen resolution set to 1024 × 768 pixels, a sampling frequency of 250 Hz, and a spatial resolution of 0.03 degrees. Participants were seated in a dark, soundproof room, 65 cm from a 22-inch widescreen LCD monitor, with their vision centered on the display. Data inclusion required successful completion of a five-point calibration and the full experiment, retaining only data from compliant participants.

#### 2.2.3 Stimuli

Eye tracking stimuli consisted of two videos previously used in related research (13, 14). The first video (25 seconds) featured a woman and a tablet, alternating attention between the two based on the tablet’s activity (turning on/off), testing joint attention capabilities. The second video (10 seconds) displayed a woman silently mouthing the alphabet, focusing on the eyes and mouth as separate areas of interest (AOIs) to examine social communication and early language development cues (18). These two videos were specifically designed and selected to test ASD-related core deficits such as social referencing and contextual understanding of social environments.

#### 2.2.4 Autism diagnostic observation schedule

ADOS-2, the gold standard in ASD diagnostics, involves several modules selected based on the participant’s age and language development, assessing social interactions, communication, and behaviors (6, 26, 27). It ends with a diagnostic algorithm tailored to maximize diagnostic sensitivity and specificity. Each module’s outcomes are quantified into a calibrated severity score (CSS) from 1 to 10 (28). In the present study, the ADOS-2 modules T, 1, and 2 were utilized and their administration involved two formally trained professionals for ADOS-2; additionally, three trained professionals, each with 1-2 years of specialized experience, performed the eye tracking data collection. The overall evaluation process takes approximately one hour.

### 2.3 Statistical analysis

Eye tracking raw data was processed using Tobii Pro software. Consistent with prior studies, data segments with less than 25% screen-looking time were excluded, as were participants with fewer than 50% valid trials (29–31). Comparisons of TGC, ASC, FAS, and AVC between ASD and non-ASD groups utilized the Wilcoxon rank-sum test, while discriminant analysis evaluated the ability of AOIs to categorize subjects by ASD severity. Detailed AOI shift analysis within and across different attention time blocks for video 1 was performed. Correlations between TGC, ASC, FAS, AVC, and ADOS-2 total/sub-scores were examined using R (version 4.4.1) to determine the sensitivity and specificity of ET metrics in predicting ASD diagnosis, identifying optimal cutoff scores for effective separation of ASD and non-ASD groups. The optimal ADOS-2 cutoffs were determined independently by two domain experts independently, both of which reported a consistent cutoff of 5 as the optimal value.

## 3 Results

### 3.1 Participant demographics

For this study cohort, 39 high-risk individuals for ASD were included in data analysis. The participants included 22 ASD individuals and 17 non-ASD individuals. Among them 25 males and 14 females; 15 White (42.8%), 11 Asian (31.4%), and 9 (25.7%) subjects of other races. **Table 1** shows the demographic and clinical features of all the participants. There were no significant differences in age or gender between ASD and non-ASD in the two groups; however, their ADOS-2 total, and sub-scores were significantly different as expected (**Table 1**).

**Table 1:** Summary of study participant demographics and ADOS-2 scores.

	ASD [Mean (SD)]	Non-ASD [Mean (SD)]
Age (years)	3.8 (1.66)	3.3 (1.77)
ADOS-2 Scores		
Total	7.77 (1.8)	4.47 (2.32)
Social Affect	8 (1.72)	5.41 (2.9)
Restrictive and Repetitive Behavior	7.09 (1.97)	3.18 (2.24)
Sex (n)		
Male	16	9
Female	6	8
Race/Ethnicity (n)		
White	8	9
African American	2	0
Asian	7	6
Hispanic	3	0
Multi-racial	2	2

**Figure 1:**
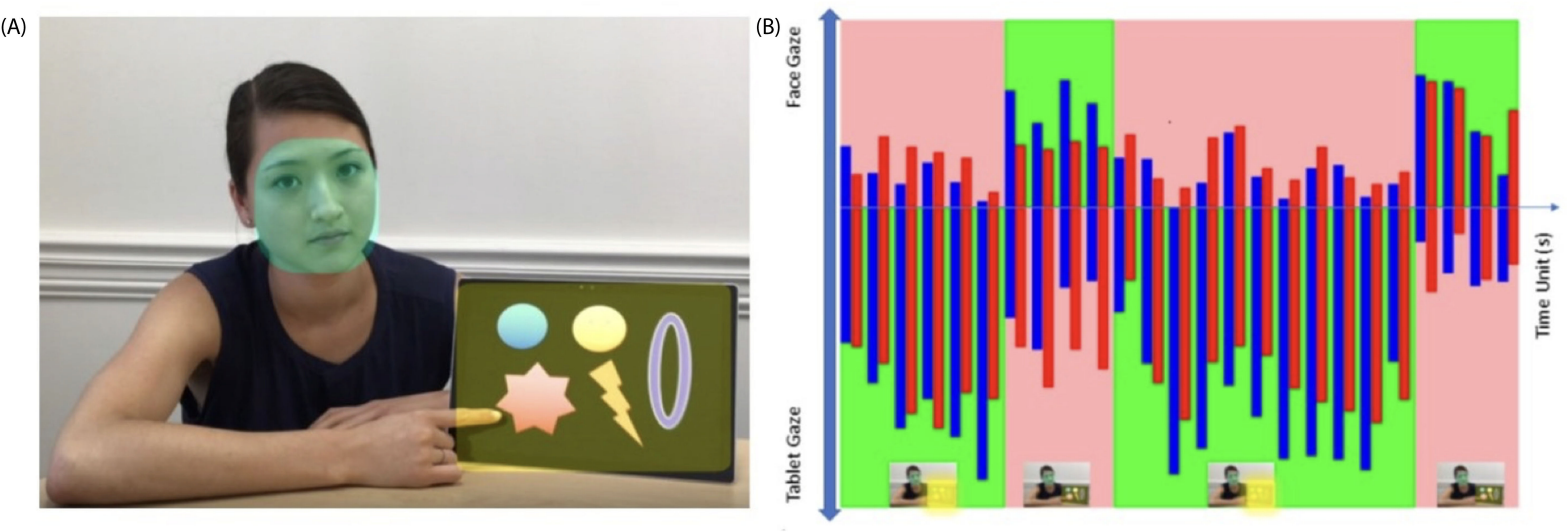
**(A)** Video 1, AOI 1 (face) and AOI 2 (tablet); **(B)** mean gaze numbers in each second (totally 25s). Blue bars: non-ASD group; Red bars: ASD group. Green areas: Favored AOI Shift (FAS). Pink areas: Unfavored AOI shift (UAS).

The difference of FAS and AVC between ASD and non-ASD individuals. Video 1 (25s) contains a woman (AOI-1 is her face) on the left side of the screen and a tablet (AOI-2) on the right side of the screen (**Figure 1A**). The video elapsed a total of 25s divided into four blocks of time 1-2-3-4 as described above in the protocol (**Figure 1B**): Block 1 is when the tablet is on with pictures moving, meant to draw subjects’ attention to watch; Block 2 is when the woman suddenly turns off the tablet, and we expect subjects to turn and look at the woman’s face, wondering what is going on at this point; Block 3 is when the woman turned on the tablet again; and Block 4 is when the woman turns off the tablet again. The attention shifts were expected during tablet on-off-on-off. The blue bars represent the TGC of non-ASD group, the red bars represent the TGC of ASD group; green colored areas are FAS pathway which are expected normally subjects would do following the sequence of tablet-face-tablet-face vs the opposite. Pink colored areas are unfavored attention shift (UAS) showed on **Figure 1B**.

The TGC was analyzed for both ASD and non-ASD groups in two AOIs cross the different time blocks (**Table 2**). We found that non-ASD individuals showed significant TGC differences cross time blocks 1→2, 2→3 and 3→4 for both AOI areas. Instead, ASD children had no TGC difference during 1→2 and 2→3 shifts, and only started to show difference during 3→4 shift for both AOI areas; meanwhile the difference between both subject groups showed significance 1→2 and 2→3 but not 3→4 (**Table 2**). When we further investigated FAS pathway and AVC which is subject’s gaze counts on neither AOIs, we found that ASD group had significantly reduced TGC along FAS (*p* < 0.00001) and significantly increased in AVC (*p* < 0.00001) across different time blocks relative to non-ASD group (**Table 3**).

**Table 2:** The comparison of significance of total gaze counts cross time blocks in ASD vs non-ASD groups.

Time Block	AOI	Intra-time block *P*-value		Groupwise *P*-value
		Non-ASD	ASD	
1 → 2	Face	0.000011	0.8261	0.00554
	Tablet	0.000055	0.1004	0.1508
2 → 3	Face	< 0.00001	0.1491	0.01682
	Tablet	0.000033	0.9966	0.01735
3 → 4	Face	< 0.00001	0.000733	0.5203
	Tablet	< 0.00001	0.000039	0.1704

**Table 3:** The comparison of favored shifts and vacant attentions in ASD vs non-ASD groups.

		Non-ASD TGC	ASD TGC	*P*-value
AOI	Face	314. ± 608.	288. ± 651.	0.5249
	Tablet	995. ± 1030.	789. ± 1043.	0.002142
FAS		1022. ± 1039.	737. ± 1022	< 0.00001
FAS-UAS		735. ± 1281.	396. ± 1341.	0.00007
AVC		4.53 ± 9.64	9.18 ± 12.1	< 0.00001

### 3.2 The difference of ASC and AVC between ASD and non-ASD individuals

**Figure 2:**
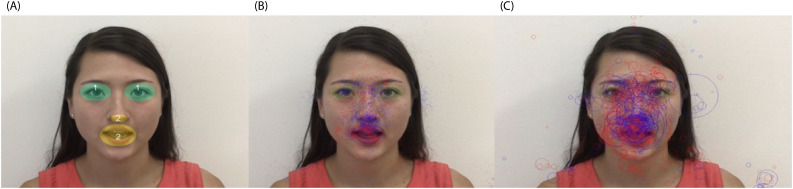
Video 2 shows a woman mouthing the alphabet without sound **(A)** AOI-1 for both eye areas (green areas), and AOI-2 for nose and mouth area (yellow areas). **(B)** distribution of total gaze counts (TGG). **(C)** distribution of total fixation time (TFT). Blue dots: non-ASD group, red dots: ASD group.

Video 2 (10 seconds) consisted of a woman sitting and mouthing the alphabet without sound. We defined two important AOIs: AOI-1 was defined as the eye area and AOI-2 was defined as the mouth area (**Figure 2**); We studied their TGC in these AOIs, and ASC which are the switches between these two AOIs. Red dots represent the ASD group and blue dots represent non-ASD group. **Figure 2B** shows TGC and **Figure 2C** showed TFT for both groups. We can see the different density distribution pattern between the ASD and the non-ASD groups. The ASD group has a more diverse and scattered distribution.

When we investigated the detailed TGC, ASC, and AVC, we found that the ASD group had significantly less ASC between AOI 1 and 2 (*p* = 0.0452), and significantly more AVC (*p* = 0.000017) vs the non-ASD group, while TGC was found to be significantly smaller in the ASD group than the non-ASD group for AOI-1 area (*p* = 0.00379), not AOI-2 area (*p* = 0.6537). We also compared with the old EP metrics TFT and pupil size, they all showed no significant difference between two groups (*p* > 0.05), demonstrating that AVC and ASC had significantly higher sensitivity than the old metrics (**Table 4**).

**Table 4:** Comparison of old and new eye tracking metrics in ASD vs non-ASD group for video 2.

		Non-ASD	ASD	*P*-value
Eye (AOI-1)	Total gaze count (TGC)	133.3	76.0	0.00379
	Total fixation time (TFT)	91.24	56.96	0.0661
	Pupil Size	3.316	3.456	0.7485
Mouth (AOI-2)	Total gaze count (TGC)	194.5	180.4	0.6537
	Total fixation time (TFT)	163.1	159.2	0.902
	Pupil Size (mm)	3.475	3.142	0.474
ASC	Between AOIs 1and 2	5.94	4.23	0.0452
AVC	Total gaze counts	1.812	3.950	0.000017

### 3.3 Correlation of significant new ET metrics and ADOS-2 scores/ASD diagnosis

**Figure 3:**
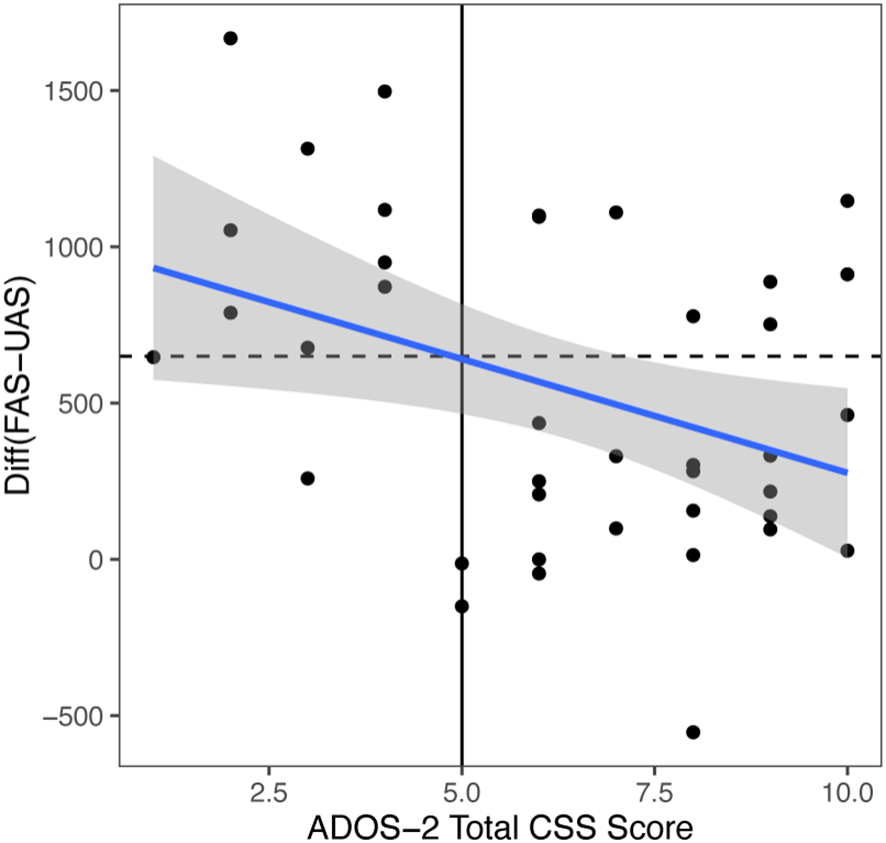
Video 1 (Face and Tablet) FAS-UAS vs ADOS-2 total scores and the linear regression fit. Linear fit: gaze = -72.841*ADOS-2 total scores + 1005.3. Correlation = -0.373. If ADOS-2 total scores cutoff = 5, the gaze cutoff was calculated as: gaze cutoff = 641.1 (below would be diagnosed), specificity = 0.91, sensitivity = 0.72, *p* = 0.01948.

We conducted a correlation study with regression analysis between the significant ET index and ASD severity based on ADOS-2 scores. We found that FAS-UAS for video 1 negatively correlated with ADOS-2 total scores (*r* = -0.373, *p* = 0.01948), SA scores (*r* = -0.33, *p* = 0.0412) and RRB scores (*r* = -0.25, *p* = 0.124). When we use ADOS-2 total CSS cut off score 5 and FAS-UAS cut off score 641.1, we got sensitivity 91%, specificity 72% (**Figure 3**).

**Figure 4:**
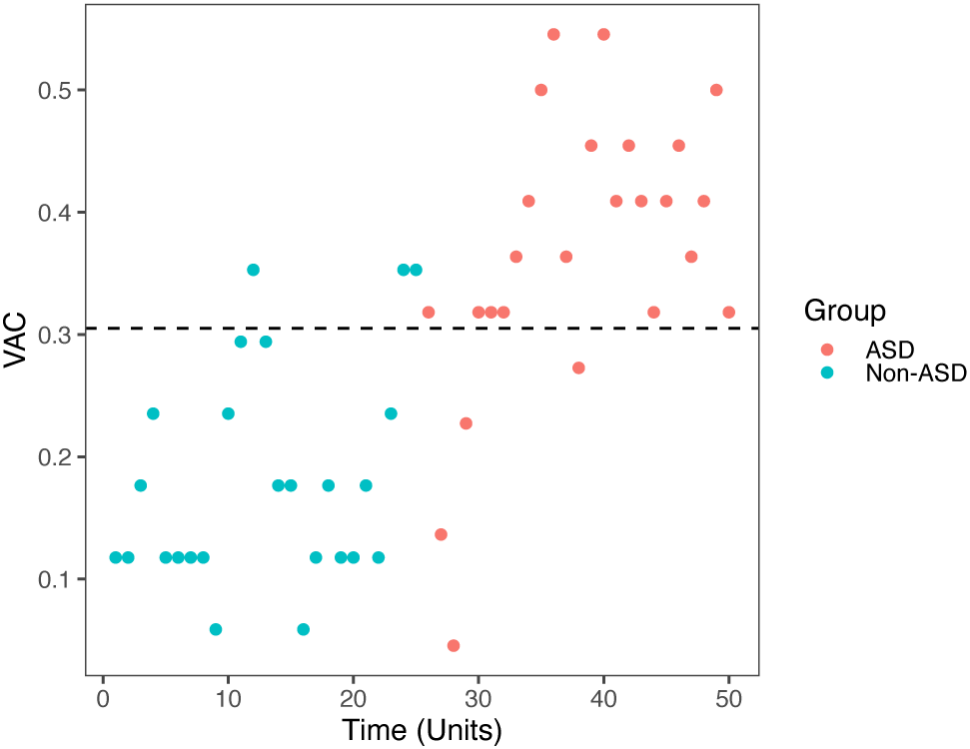
Video 1 (Face and Tablet) VAC vs time unit distributions for non-ASD (round) and ASD (triangle). Results based on the cutoff effect: cutoff = 0.305 (above would be diagnosed), Specificity = 0.88, Sensitivity = 0.88, *p* < 0.00001.

**Figure 5:**
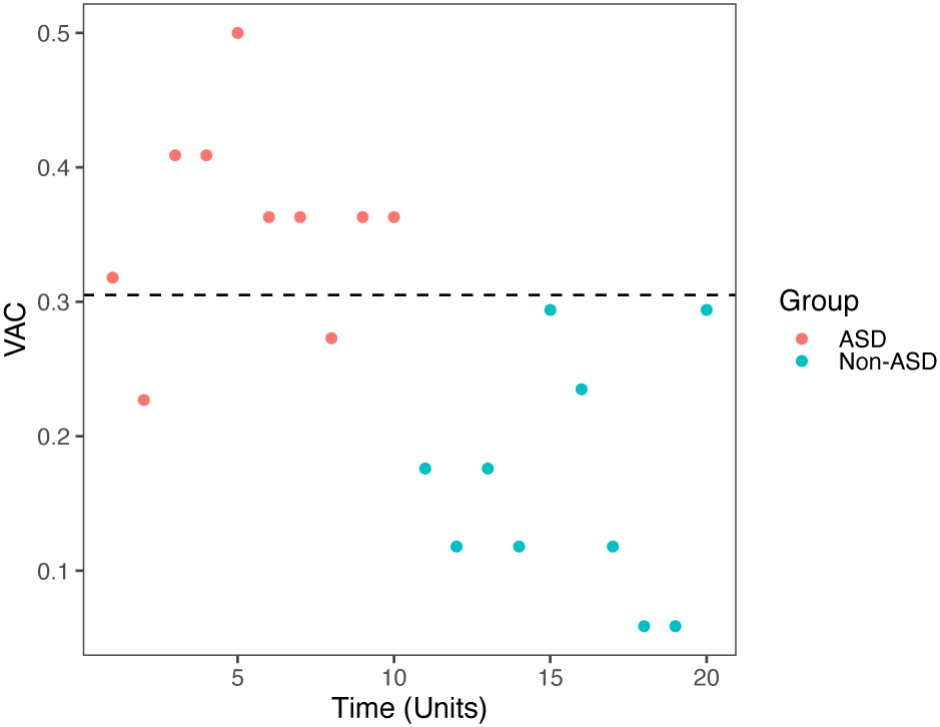
Video 2 (Speaking) VAC vs time unit distributions for non-ASD (round) and ASD (triangle). Results based on the cutoff effect: cutoff = 0.306 (above would be diagnosed), specificity = 1.00, sensitivity = 0.80, *p* = 0.000045.

When we compared ASD (red dots) vs non-ASD (blue dots) group, we found that AVC had the best sensitivity and specificity among all the ET metrics (new or old), the results were consistent in both video 1 (sensitivity 88%, specificity 88%, *p* < 0.00001, cut-off score 0.305) showed in **Figure 4**, and video 2 (sensitivity 100%, specificity 80%, *p* < 0.000045, cut-off score 0.306) in **Figure 5**. ASC for video 2 had sensitivity 71%, specificity 64%, *p* = 0.04523, and cut off score 4.5.

## 4 Discussion

Previous investigations into ET as a diagnostic tool for ASD have shown promising results, such as notable reductions in TFT of AOIs among ASD subjects (13, 19). However, the diversity in ET protocols and paradigms has often limited its reliability as a diagnostic instrument (23). For example, the traditionally used ET metrics such as TFT have limited sensitivity and specialty in differentiating between ASD and non-ASD individuals. Nonetheless, ongoing research have introduced metrics that achieves higher accuracies for the diagnosis of ASD (25). Given these advancements, our study aims to explore alternatives to the previous efforts through the discovery of a novel set of ET metrics—FAS, ASC, and AVC—designed to enhance diagnostic accuracy by capturing subtle behavioral markers fundamental to ASD.

The introduction of FAS versus UAS aims to differentiate between typical and atypical attention shifts, reflecting an individual’s ability to prioritize relevant stimuli dynamically. This approach underscores cognitive flexibility, a critical aspect often impaired in ASD. ASC and AVC, respectively, quantify transitions between competitive targets and the absence of gaze on expected targets, providing a nuanced understanding of attentional engagement and disengagement in ASD individuals.

Our findings indicate these new metrics are more sensitive and specific than traditional measures like TFT and pupil size, particularly in distinguishing ASD from non-ASD in a cohort of high-risk individuals. Furthermore, our findings justify an enhanced ability for the metrics in capturing ASD core symptom differences between those with and without ASD relative to previous findings. For instance, our analysis revealed that ASD participants exhibited significantly fewer FAS and heightened UAS during tasks designed to test joint attention (JA), a fundamental social communicative skill that is typically disrupted in ASD (32). Notably, the correlation of these metrics with ADOS-2 scores suggests that the severity of social affect impairments is inversely related to the engagement in favored gaze patterns.

The AVC metric emerged as particularly insightful, revealing that ASD individuals frequently failed to engage with designated AOIs—indicative of the inattentive phenomenon often reported anecdotally by caregivers of ASD individuals. The statistical robustness of AVC (sensitivity of 88-100% and specificity of 80-88% across various tests) supports its potential utility in clinical settings, emphasizing its role in detecting divergent attention patterns.

This study not only reaffirms the utility of ET in ASD diagnosis but also introduces new avenues for understanding the neural and cognitive underpinnings of the disorder. The reduced tendency of ASD individuals to shift attention as expected may reflect broader deficits in theory of mind and social cognition, potentially linked to underlying neural abnormalities in networks involving the cerebellum and prefrontal cortex (33, 34).

While our results are promising, the specificity of the participant cohort—high-risk individuals rather than a broader demographic including non-ASD individuals—necessitates cautious interpretation. Future research should aim to validate these findings across more diverse populations and clinical settings, enhancing the generalizability of the metrics. Other limitations should be noted, such as limited sample size and lack of ethnic diversity, which may limit the generalizability of our findings to a broader and more diverse population.

Despite the mentioned limitations, our study positions ET not only as a feasible diagnostic tool for early ASD screening but also highlights its potential to provide deeper insights into the distinct neurodevelopmental trajectories associated with the disorder. The development of ET metrics like FAS, ASC, and AVC marks a significant advance in the objective measurement of core ASD features, paving the way for more targeted interventions and therapies tailored to individual neurodevelopmental profiles. As we continue to refine these metrics and explore their clinical implications, large-scale studies will be essential for establishing their efficacy and integrating them into routine clinical practice for ASD screening and formal clinical diagnosis.

## Data Availability

The raw data supporting the conclusions of this article will be made available by the authors, without undue reservation.
